# Selective Attention and Sensory Modality in Aging: Curses and Blessings

**DOI:** 10.3389/fnhum.2016.00147

**Published:** 2016-03-31

**Authors:** Pascal W. M. Van Gerven, Maria J. S. Guerreiro

**Affiliations:** ^1^Faculty of Psychology and Neuroscience, Department of Neuropsychology and Psychopharmacology, Maastricht UniversityMaastricht, Netherlands; ^2^Biological Psychology and Neuropsychology, Institute for Psychology, University of HamburgHamburg, Germany

**Keywords:** aging, selective attention, sensory modality, inhibition, enhancement

## Abstract

The notion that selective attention is compromised in older adults as a result of impaired inhibitory control is well established. Yet it is primarily based on empirical findings covering the visual modality. Auditory and especially, cross-modal selective attention are remarkably underexposed in the literature on aging. In the past 5 years, we have attempted to fill these voids by investigating performance of younger and older adults on equivalent tasks covering all four combinations of visual or auditory target, and visual or auditory distractor information. In doing so, we have demonstrated that older adults are especially impaired in auditory selective attention with visual distraction. This pattern of results was not mirrored by the results from our psychophysiological studies, however, in which both enhancement of target processing and suppression of distractor processing appeared to be age equivalent. We currently conclude that: (1) age-related differences of selective attention are modality dependent; (2) age-related differences of selective attention are limited; and (3) it remains an open question whether modality-specific age differences in selective attention are due to impaired distractor inhibition, impaired target enhancement, or both. These conclusions put the longstanding inhibitory deficit hypothesis of aging in a new perspective.

Imagine an older person browsing the internet, attempting to find his or her way to a certain piece of information while trying to ignore advertisement banners, irrelevant links, sounds, and movies. You probably envision this person as being challenged, if not overwhelmed, by the multisensory streams of information in this situation. This impression is in line with the *inhibitory deficit hypothesis* (Hasher and Zacks, 1988; Lustig et al., 2007), the longstanding view that older adults have a declined ability to inhibit the processing of irrelevant, distracting information.

Yet this view is primarily based on studies that investigated selective attention within the visual modality (Figure 1A; Guerreiro et al., 2010). Only a minority of prior studies investigated selective attention within the auditory modality, while a particularly small minority investigated selective attention across sensory modalities. This is remarkable because in daily life, we commonly find ourselves in situations involving all combinations of visual or auditory relevant, and visual or auditory irrelevant information (Figure 1B).

**Figure 1:**
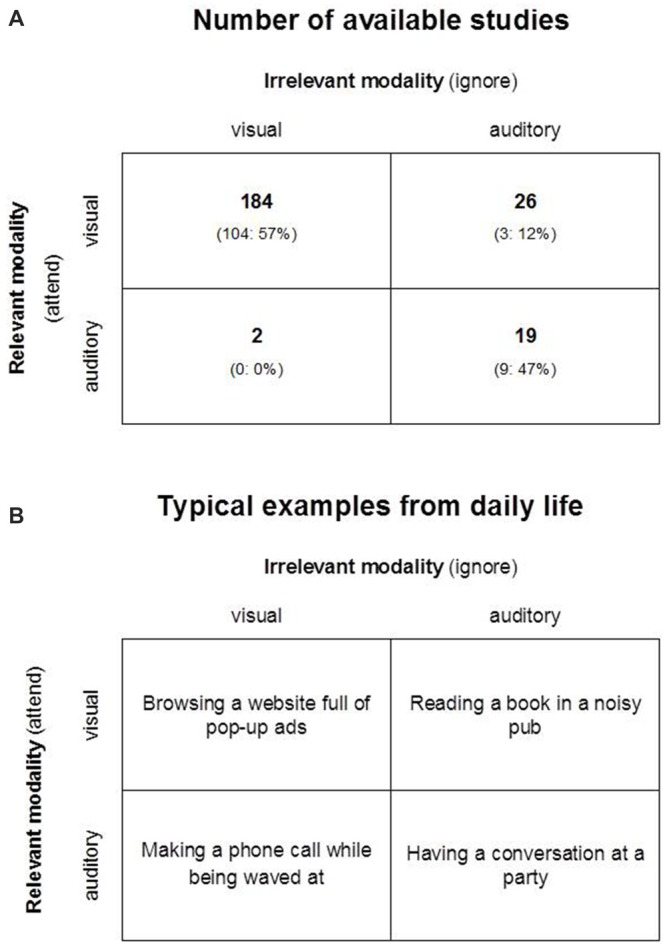
**A fully crossed scheme of selective attention as a function of sensory modality.** All possible combinations of relevant (to-be-attended) and irrelevant (to-be-ignored) sensory modalities with the number of available studies in 2010 **(A)** and typical examples from daily life **(B)**. Information in **(A)** is based on the systematic review by Guerreiro et al. (2010, Table 9, p. 1012). Numbers include individual studies in multiple-study research articles. Numbers in brackets indicate studies (number and percentage) that found support for age-related differences of selective attention.

The notion that sensory modality may crucially determine age-related differences of selective attention came from the anomalous but consistent observation that younger and older adults are equally distracted by irrelevant speech while performing a visual task. Since the seminal work on this age-equivalent *irrelevant speech effect* by Rouleau and Belleville (1996), around a dozen studies have been performed to replicate these findings in various setups. These studies have excluded such factors as lack of statistical power, age-related hearing loss (both investigated by Bell and Buchner, 2007), level of interference (Van Gerven et al., 2007b), and emotional valence of the irrelevant speech (Van Gerven and Murphy, 2010). However, none of these studies have been able to show age-related effects, and so an intriguing challenge for the inhibitory deficit hypothesis was born.

## Filling in the Blanks: Behavioral Studies

Inspired by this challenge, we performed a systematic literature review on the role of sensory modality in age-related distractibility (Guerreiro et al., 2010). From this review, it appeared that older, relative to younger, adults tend to be disproportionately distractible in circumstances where: (1) distracting stimuli are presented through the same sensory modality as target stimuli; and (2) distracting stimuli are visual rather than auditory. However, none of the studies in our review had shown this pattern of results in a *fully crossed design*, entailing each combination of visual or auditory target, and visual or auditory distractor information, with analogous tasks across conditions.

**Figure 2:**
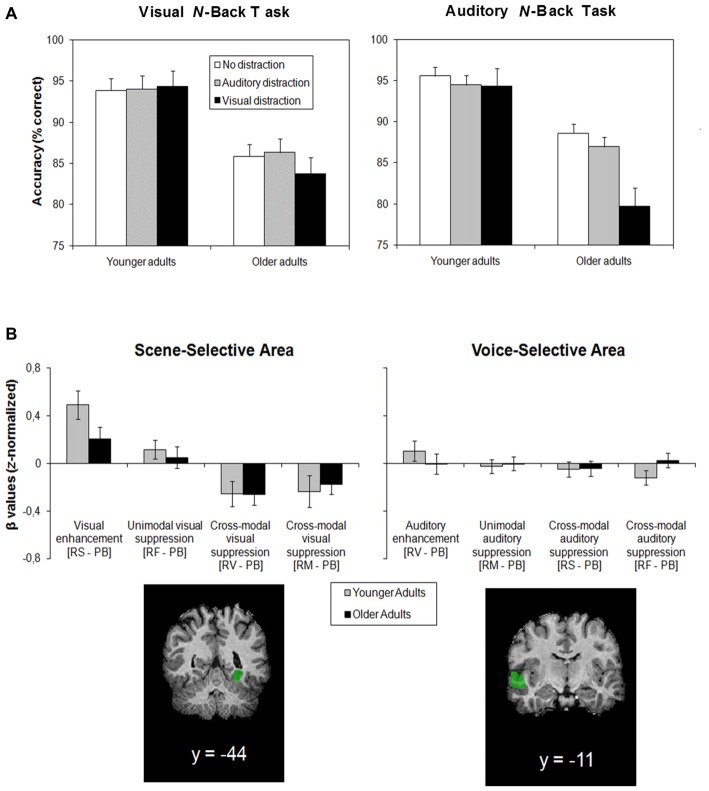
**(A)** Mean accuracy of younger and older adults performing on the visual and auditory *n*-back task (averaged over 1- and 2-back conditions, which explains the relatively high mean accuracy scores of around 80% and higher) without distraction, with auditory distraction, and with visual distraction. Error bars indicate standard errors of the mean. Adapted from Guerreiro et al. (2013), with permission from Elsevier. **(B)** Results from the adapted Gazzaley et al. (2005) paradigm. Depicted are mean enhancement and suppression effects of different attentional conditions on activity in the scene-selective area (parahippocampal place area) and the voice-selective area (temporal voice area). Positive values indicate enhancement; negative values indicate suppression (relative to a perceptual baseline: cortical activity when passively viewing the stimuli). Attentional conditions are abbreviated as follows: RS, remember scenes; RF, remember faces; RV, remember voices; RM, remember music; PB, perceptual baseline. Error bars indicate standard errors of the mean. Adapted from Guerreiro et al. (2015), with permission from Elsevier.

To fill in these blanks, we developed a fully crossed behavioral paradigm based on a numerical *n*-back task. In this task, a random sequence of digits between 1 and 9 is presented one at a time. Participants are required to match the current digit with the digit that appeared *n* digits back in the sequence. This is quite demanding, especially for older adults, and especially if *n* is raised from 1 to 2 (Van Gerven et al., 2007a). We designed a visual and an auditory version of this task. Distractors were concurrently presented irrelevant digits. Targets and distractors were superimposed onto each other in different colors in the unimodal visual condition, concurrently presented in different voices through a set of headphones in the unimodal auditory condition, or concurrently presented through different sensory modalities in the cross-modal conditions. These conditions were compared with a control condition without distraction. Using this paradigm in two independent studies (Guerreiro and Van Gerven, 2011; Guerreiro et al., 2013), we found that performance accuracy of older participants was compromised only in the auditory *n*-back task with visual distraction. The visual *n*-back task with auditory distraction and the unimodal tasks did not yield any age-related differences in distraction (Figure 2A).

We have sought to replicate these findings in two tasks with a spatial component. In both of these tasks the location of a cue or distractor—which could be presented left or right—either did or did not correspond with the location of the target stimulus (target localization task; Guerreiro et al., 2012) or the location of the relevant response (i.e., left or right index finger in a response interference task; Guerreiro et al., 2014a). Again, we developed visual and auditory versions of these tasks with visual, auditory, or no distraction. Although the cue or distractor location was completely irrelevant to the tasks, corresponding locations tended to speed up performance, whereas non-corresponding locations tended to slow performance down. However, these effects did not differ across age groups, suggesting that the aforementioned modality-specific age effects do not extend to spatial selective attention tasks.

## Filling in More Blanks: Psychophysiological Studies

The role of sensory modality in age-related selective attention has been more extensively explored in the psychophysiological literature. Studies in this field have focused on the modulation of modality-specific brain activity during cross-modal selective attention: attending to stimuli presented through the visual or auditory modality while ignoring stimuli from the other modality. Remarkably, most of these studies have not yielded any age differences (see Peiffer et al., 2009, for an exception). For example, Hugenschmidt et al. (2009) found that both enhancement of task-relevant, and suppression of task-irrelevant modality-specific cortical activity was age independent. Also ERP studies by Mishra and Gazzaley (2013) and by Guerreiro et al. (2014b) did not yield age differences in cortical activity driven by cross-modal selective attention.

Importantly, none of the aforementioned studies covered the fully crossed scheme depicted in Figure 1A. We recently attempted to resolve this issue with an fMRI study (Guerreiro et al., 2015), involving an extension of a task developed by Gazzaley et al. (2005), which originally only covered the visual modality. The idea behind this task is that the processing of distinct categories of stimuli takes place in distinct, category-selective, parts of the cortex. The activity of these cortical areas depends on the instructions given to the participant: if stimuli from a particular category (e.g., scenes), presented among a random sequence of stimuli from another category (e.g., faces), need to be attended and remembered a few seconds later, then the activity in the corresponding cortical area (i.e., parahippocampal place area) should be *enhanced* relative to baseline (i.e., activity when passively viewing the same stimuli, without memorizing them). If, on the other hand, the same stimuli (i.e., scenes) need to be ignored, the activity in the corresponding area (i.e., parahippocampal place area) should be *suppressed*. Using this paradigm, Gazzaley et al. (2005) found older adults to be impaired in cortical suppression of distraction during unimodal visual attention.

We extended Gazzaley et al. (2005) paradigm such that it included auditory and cross-modal selective attention conditions. For that purpose, we defined four categories of stimuli: two visual categories, faces and scenes; and two auditory categories, voices and music sounds. This enabled us to investigate both unimodal selective attention—by measuring brain activity related to two stimulus categories from the same modality—and cross-modal selective attention—by measuring brain activity related to two stimulus categories from different modalities.

We confined our analyses to two stimulus categories, scenes and voices, because their corresponding cortical areas, the parahippocampal place areas and the temporal voice area, appear the most robust markers of top-down modulation (e.g., Bestelmeyer et al., 2011; Chadick et al., 2014). Figure 2B shows how these areas behaved in younger and older adults in different attentional conditions. Whereas no significant modulation of activity in the voice-selective area was found in either age group, activity in the scene-selective area was more telling: it revealed age-equivalent enhancement of visual target information (i.e., in both age groups, activity in the scene-selective area was higher when scenes were attended than when scenes were passively observed), as well as—and most importantly—age-equivalent suppression of cross-modal visual distraction (i.e., in both age groups, activity in the scene-selective area was lower when ignoring scenes to attend to auditory stimuli than when passively observed). Together with earlier findings of age-equivalent suppression of cross-modal auditory distraction (e.g., Guerreiro et al., 2014b), this suggests that cross-modal inhibition is robust against aging.

## Some Methodological Considerations

From our concise but representative review, it appears that age-related differences in selective attention is by no means self-evident: its dependency on multiple factors—most notably, sensory modality—suggests that selective attention in older age is cursed and blessed at the same time. There are, however, some important methodological factors that should be taken into account when evaluating the variety of results from the aforementioned studies (for exhaustive overviews, see Guerreiro et al., 2010; Zanto and Gazzaley, 2014).

First, one should be aware of the diverse contexts in which the term “selective attention” is used. We define selective attention as the process that deals with situations in which there are one or more target stimuli, or stimulus dimensions (e.g., size, color, location), among one or more distractor stimuli, or stimulus dimensions, in close spatial and temporal proximity, which retain their role for the duration of the task. This definition corresponds to the “access control” function of inhibition, as defined by Friedman and Miyake (2004), and excludes the “deletion” function, which reduces proactive interference. The latter is known to be impaired in older age, as has been shown, for example, in task-switching paradigms (e.g., Wasylyshyn et al., 2011; Lawo et al., 2012).

Second, the nature of the distracting information in the different paradigms needs to be considered. Distractors may interfere with target stimuli either because of their identity or because of their location. Identity-based interference takes place at the perceptual level, whereas location-based interference may take place at both the perceptual and the response level. We have shown earlier that modality-specific age differences in selective attention only occur at the perceptual level.

A third factor to take into account is the timing and frequency of the distracting information. Distractors can be presented either serially—that is, before the targets—or concurrently—that is, together with the targets. The strength of the distraction in “serial” tasks may be weaker than in “concurrent” tasks, which could be the reason why no age-related effects were found with the target localization task employed by Guerreiro et al. (2012). In a related vein, the frequency at which targets and distractors are presented may determine the modulatory effects in the corresponding cortical areas. A relatively low frequency of target and distractor presentation may explain why we found no significant attentional modulation of the voice-selective area (Guerreiro et al., 2014a), whereas others did (e.g., Salo et al., 2015). Because we did find significant modulation of the scene-selective area at the same stimulus frequency, however, this may imply that different cortical areas require different stimulus frequencies to elicit a measurable modulatory response.

A fourth and final factor to consider is task difficulty (Zanto and Gazzaley, 2014). It is well known that age differences tend to increase with increasing task difficulty (e.g., Salthouse, 1992). However, in our *n*-back studies (Guerreiro and Van Gerven, 2011; Guerreiro et al., 2013), task difficulty did not affect the pattern of modality-dependent age effects. Although increasing *n* from 1–2 strongly increases the coordinative complexity of this task,^1^ especially for older adults, who showed a larger drop in accuracy in the 2-back relative to the 1-back condition (cf. Verhaeghen and Basak, 2005; Van Gerven et al., 2008), this neither influenced the distractibility of the younger, nor did it influence the distractibility of the older participants (therefore, *n*-back task performance is collapsed over 1 and 2-back conditions in Figure 2A). On the one hand, this result is at odds with neuroimaging studies showing that—in younger adults—auditory distraction is suppressed when the load imposed by the primary visual task is low, but not when it is high (Gisselgård et al., 2003, 2004). On the other hand, our result is in line with the findings by Rees and colleagues (Rees and Lavie, 2001; Rees et al., 2001), who have demonstrated—again, in younger adults—that visual distraction is processed regardless of the load imposed by the primary auditory task.

Task difficulty may also vary with the distribution of information over sensory modalities, making unimodal tasks more difficult than cross-modal tasks because unimodal tasks involve a higher perceptual load (Brand-D’Abrescia and Lavie, 2008). However, this is not in line with our observations that cross-modal auditory selective attention is affected by aging, whereas cross-modal visual selective attention—where perceptual load is the same—and unimodal selective attention—where perceptual load should be higher—are not.

## Conclusions and Future Directions

Based on our recent findings, our current conclusions are threefold. First, age-related deficits of selective attention are modality dependent. That is, relative to younger adults, older adults are disproportionately disadvantaged when they are engaged in an auditory task with visual distraction, not in the reversed situation or in situations where targets and distractors are presented through the same sensory modality. This modality-dependent pattern of results does not extend to spatial selective attention tasks. Second, age-related deficits of selective attention are limited. In comparable tasks and distraction settings across sensory modalities, we only see age effects in one combination of sensory modalities and only in a non-spatial task. Third and finally, it is currently unresolved whether modality-specific age-related differences of selective attention are primarily due to impaired inhibition—the usual suspect, impaired enhancement, or both. In fact, a number of psychophysiological studies, including our own, have shown that both enhancement of target processing and inhibition of distractor processing are intact, especially in cross-modal situations. This may foreshadow yet another challenge for the inhibitory deficit hypothesis.

So, where to from here? First and foremost, the neural mechanisms underlying the modality dependence of age-related selective attention observed at the behavioral level should be determined. Possibly, a relatively weak, but normal, modulation of the auditory cortex makes cross-modal auditory selective attention particularly vulnerable to age-related decline. Because in healthy aging, neural changes may not be extensive enough to detect such vulnerability, it is of particular interest to investigate attention-driven modulation of cortical activity in pathological aging. Especially individuals with incipient or progressed dementia of the Alzheimer type (DAT), who show pronounced impairments of selective attention relative to healthy older adults (Levinoff et al., 2004; Deiber et al., 2009; Coubard et al., 2011), are an interesting target group. For example, Golob et al. (2001) have demonstrated disrupted cross-modal suppression of visual cortical processing in DAT patients, which is in line with our own observation that auditory task performance during visual distraction is impaired in healthy older adults (e.g., Guerreiro and Van Gerven, 2011). Finally, Jacobs et al. (2012) have pointed out that early-stage DAT patients especially show profound atrophy in the parietal cortex, which is strongly involved in selective attention. Changes and individual differences in structural integrity of the parietal cortex, such as indicated by cortical thickness, may therefore be predictive of selective attention performance in both healthy and pathological aging (see, e.g., Chadick et al., 2014, for similar relations between medial frontal cortex integrity and age-related distractibility). We realize, however, that healthy and pathological aging are not necessarily part of the same continuum. Although it has been shown that both cognitive and neural impairment in DAT are quantitatively, rather than qualitatively, different from normal aging (e.g., Walters, 2010; Serrano-Pozo et al., 2013), there are also studies showing the opposite (e.g., Ohnishi et al., 2001). Therefore, caution should be taken in the hypothesized outcomes of future studies on modality-related selective attention in DAT.

As soon as the modality dependence of age-related selective attention has been determined at both the behavioral and the neural level, a next step would be to investigate whether it can be altered. Mozolic et al. (2011) have shown that selective attention in different sensory modalities can be improved in healthy older adults through an intensive training with visual and auditory tasks combined with unimodal or cross-modal distraction. Moreover, this improvement appears to transfer to related cognitive domains, such as dual-task performance, which is a rarity in research on protective effects of cognitive training against age-related decline (see, e.g., Salthouse, 2006).

A final interesting direction relates to the phenomenon that older adults tend to show increased levels of multisensory integration, possibly to compensate for unimodal perceptual decline (Laurienti et al., 2006; Diaconescu et al., 2013). This tendency may be useful to turn the age-related deficit in auditory cross-modal selective attention into a benefit by presenting irrelevant but *congruent* visual information during the auditory task (Weeks and Hasher, 2014). Such effects have already been found in healthy older adults during text comprehension (Kim et al., 2007) and cross-modal speech perception (Tye-Murray et al., 2011). This potentially bright side of age-related distractibility may open the way for improved information design and novel clinical interventions to optimize attentional control in both healthy and pathological aging.

## Author Contributions

PWMVG and MJSG contributed equally to this work.

## Funding

Part of our work is funded by the Netherlands Organization for Scientific Research (NWO, Grant no. 406-14-057).

## Conflict of Interest Statement

The authors declare that the research was conducted in the absence of any commercial or financial relationships that could be construed as a potential conflict of interest.

## Footnotes

^1^Verhaeghen and Basak (2005) have pointed out that increasing *n* from 1 to 2 introduces the requirement to shift focal attention in working memory with every new stimulus that is presented. This requirement also applies to values of *n* that are greater than 2. Thus, increasing *n* from 1 to 2 introduces a major increment in task difficulty, which is not seen if *n* is further increased.
